# Macroinvertebrate Communities Vary With Surface Water Permanence but Not Land Management in a Tallgrass Prairie Stream Network

**DOI:** 10.1002/ece3.73289

**Published:** 2026-03-19

**Authors:** Olivia Tow, Megan C. Malish, Stephen C. Cook, Michael T. Bogan, Daniel C. Allen, Thomas M. Neeson

**Affiliations:** ^1^ Department of Geography and Environmental Sustainability University of Oklahoma Norman Oklahoma USA; ^2^ Department of Biology University of Oklahoma Norman Oklahoma USA; ^3^ School of Natural Resources and the Environment University of Arizona Tucson Arizona USA; ^4^ Department of Ecosystem Science and Management Pennsylvania State University, State College University Park Pennsylvania USA

**Keywords:** aquatic macroinvertebrates, biodiversity, grazing, land management, nonperennial streams, prescribed fire, streamflow permanence, tallgrass prairie

## Abstract

Native tallgrass prairies once covered much of the North American Great Plains but have largely been converted to agriculture and other land uses. The remaining areas of native prairie thus serve as critical benchmarks for understanding global change. Konza Prairie Biological Station (KPBS) is located within one of the largest remaining areas of native tallgrass prairie. Streams within the biological station are characterized by hydrologic extremes, including prolonged periods of drying in some reaches. Additionally, experimental grazing and prescribed burn treatments vary among sub‐watersheds. To examine the effects of surface water permanence, grazing, and prescribed burns, we collected stream aquatic macroinvertebrate samples from 10 sites annually for 2 years. We targeted both benthic and edge aquatic macroinvertebrate communities in our sampling. We also used continuous logger data from each site to classify sites as one of three stream types: short‐flowing, long‐flowing, or perennial. We expected aquatic macroinvertebrate richness and diversity to increase with surface water permanence and to be greater at sites located in sub‐watersheds where no grazing or prescribed burns occur. Our analysis showed that benthic and edge macroinvertebrate richness varied with stream type and sampling year. Richness tended to increase with surface water permanence and was greater in the first year of sampling. The composition of benthic and edge communities also varied with stream type and sampling year. Macroinvertebrate diversity did not vary with any of the variables considered. Additionally, we found no impact of grazing or burn regime on aquatic macroinvertebrate community richness, diversity, or composition. In many parts of the Great Plains, streamflow is not sufficiently protected, and the spatial and temporal extent of stream drying is expanding. Our work underscores the importance of protecting streamflow in the Great Plains and that doing so is likely to benefit stream ecosystems even in the presence of potential co‐occurring disturbances.

## Graphical abstract

Konza Prairie Biological Station, located within one of the largest remaining areas of native tallgrass prairie, is unique in that grazing and prescribed burn treatments, as well as surface water permanence, vary spatially. We found that aquatic macroinvertebrate communities vary with streamflow permanence, but not grazing presence or prescribed burns treatments. Research that takes place in remaining native prairies, like Konza Prairie Biological Station, is critical to inform the conservation of streams in these rare, native ecosystems as well as the restoration of prairie streams that are more impacted.

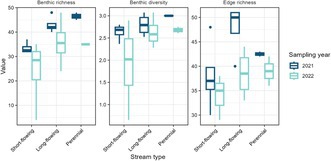

## 1 Introduction

The once widespread native tallgrass prairies of the Great Plains have been largely eliminated by human actions. It is estimated that more than 90% of the native tallgrass prairies of North America, thought to have at one time covered 67.6 million ha, no longer exist (Samson and Knopf 1994). The decline of native prairies is largely attributable to their conversion to agricultural and other land uses (Samson et al. 2004). Research that takes place in remaining native prairies is critical to inform the conservation of these rare, native ecosystems and the restoration of prairie ecosystems that are more impacted. Native tallgrass prairies can serve as a benchmark to understand the consequences of the environmental change that is taking place throughout the Great Plains region.

The loss of native tallgrass prairie ecosystems has contributed to the degradation of streams in the Great Plains. Streams that flow through remaining native prairies continue to be affected by both natural and anthropogenic disturbances (Dodds et al. 2004). Extreme hydrological events are one type of disturbance experienced by Great Plains streams. Streams in this region are characterized by harsh, flashy flow regimes. Stream drying is common, even in years with normal precipitation (Dodds et al. 2004). Drying impacts stream ecosystems in a number of ways. Early in the drying process, connections to riparian habitats are lost, removing access to habitats that are important for many organisms (Boulton 2003). As drying progresses, flowing reaches contract until isolated, non‐flowing pools form. Isolated pools typically have high nutrient concentrations, variable temperatures, and limited dissolved oxygen availability (Lake 2011). Eventually during stream drying, surface water may be lost altogether. As environmental conditions become harsher as drying progresses, taxa that cannot tolerate such conditions are lost (Bogan et al. 2015). Despite their dynamic and often harsh conditions, non‐perennial streams support diverse biological communities. Aquatic organisms in non‐perennial streams often are able to persist during drying events (i.e., resistance) or rapidly recolonize streams following drying events (i.e., resilience; Fritz and Dodds 2004). Despite the presence of resistant and resilient taxa, many studies have shown that local diversity of stream ecosystems tends to increase with increasing flow permanence (Fritz and Dodds 2005; Tornés and Ruhí 2013; Datry, Larned, and Tockner 2014; Soria et al. 2017). This pattern occurs because drying acts as a disturbance event and removes organisms (Leigh and Datry 2017).

Prairie streams are also impacted by anthropogenic disturbances. Two common forms of land management in tallgrass prairies are grazing and prescribed burns. Both function to mitigate woody encroachment by limiting the growth of woody plants (Sankaran et al. 2005). While critical to the maintenance of native prairie ecosystems, grazing and burning in prairie ecosystems can also disturb prairie streams. Activity by grazers can alter stream morphology and water quality (O'Callaghan et al. 2019). Treading and overgrazing can result in reduced stream bank stability, increased sedimentation, and widening of stream channels (Kauffman et al. 1983; Zaimes and Schultz 2011; Grudzinski and Daniels 2018). Grazers are also sources of nutrient input through urination and defecation both in the stream and throughout the watershed (Line et al. 2000; Vidon et al. 2008). As a result of the impacts to stream morphology and water quality, grazing can degrade stream habitats (Wohl and Carline 1996; Strand and Merritt 1999). Impacts of grazing can depend on factors that include grazer density, grazer access to streams, and the species of the grazer (Bilotta et al. 2007; O'Callaghan et al. 2019). For example, one study in a Great Plain tallgrass prairie found that cattle preferred riparian areas more than bison, and that cattle preferred areas with woody vegetation while bison avoided them (Allred et al. 2011). These differences have been associated with greater bare ground coverage and suspended sediment concentration in cattle‐grazed watersheds compared to bison‐grazed watersheds (Grudzinski et al. 2016, 2018). Grazing by both cattle and bison have been linked to reduced macroinvertebrate diversity (Meadows 2001; Braccia and Voshell 2007; Magner et al. 2008). The impacts of prescribed burns on stream ecosystems are not well understood. Fire can increase surface runoff and lead to increased sediment and nutrient inputs, resulting in stream habitat degradation (Paul et al. 2022; Morales et al. 2023). However, studies that have measured the effect of fire on stream ecological properties, including macroinvertebrate abundance, fish density, periphyton biomass, and biofilm biomass, have shown mixed impacts (Erdozain et al. 2024). For example, a meta‐analysis by Erdozain et al. (2024) showed that an approximately equal number of studies have reported decreased, increased, and non‐significant effects of fire on macroinvertebrate abundance.

In this study, we examined how surface water permanence, grazing, and prescribed burns influence stream macroinvertebrate communities in the native tallgrass prairie streams of Konza Prairie Biological Station (KPBS) in Kansas, USA. KPBS is located within one of the last remaining areas of native tallgrass prairie. Land management in KPBS, specifically grazing and prescribed burn treatments, varies among sub‐watersheds. Sub‐watershed grazing treatments include native grazers (bison), non‐native grazers (cattle), and no grazing. Sub‐watersheds are burned at intervals that range from 1 to 20 years. We leveraged the various land management treatments and spatially variable surface water permanence within KPBS to investigate their impacts on aquatic macroinvertebrate communities. We hypothesized that (H1) aquatic macroinvertebrate communities would be more diverse at sites with greater surface water permanence because fewer taxa are be able to persist at sites with prolonged drying events and (H2) that macroinvertebrate communities would be less diverse at sites located within sub‐watersheds with grazing and prescribed burns due to high sediment and nutrient inputs at these sites.

## 2 Methods

### 2.1 Study Area

Konza Prairie is a native tallgrass prairie ecosystem within the Flint Hills. The Flint Hills region includes the largest remaining areas of intact, native tallgrass prairie in the Great Plains. While much of the grasslands of the Great Plains were converted to agriculture, the Flint Hills area was never plowed due to its steep slopes and shallow, rocky soils. The region has a humid continental climate, characterized by distinct seasons with hot summers and cold winters. Summers are hot and dry, with average high air temperatures ranging from 30°C to 35°C, occasionally reaching above 38°C. Winters are cold, with average high air temperatures from 0°C to 10°C. The average annual rainfall is 825 mm and occurs primarily from spring to early summer (National Centers for Environmental Information 2001).

**FIGURE 1:**
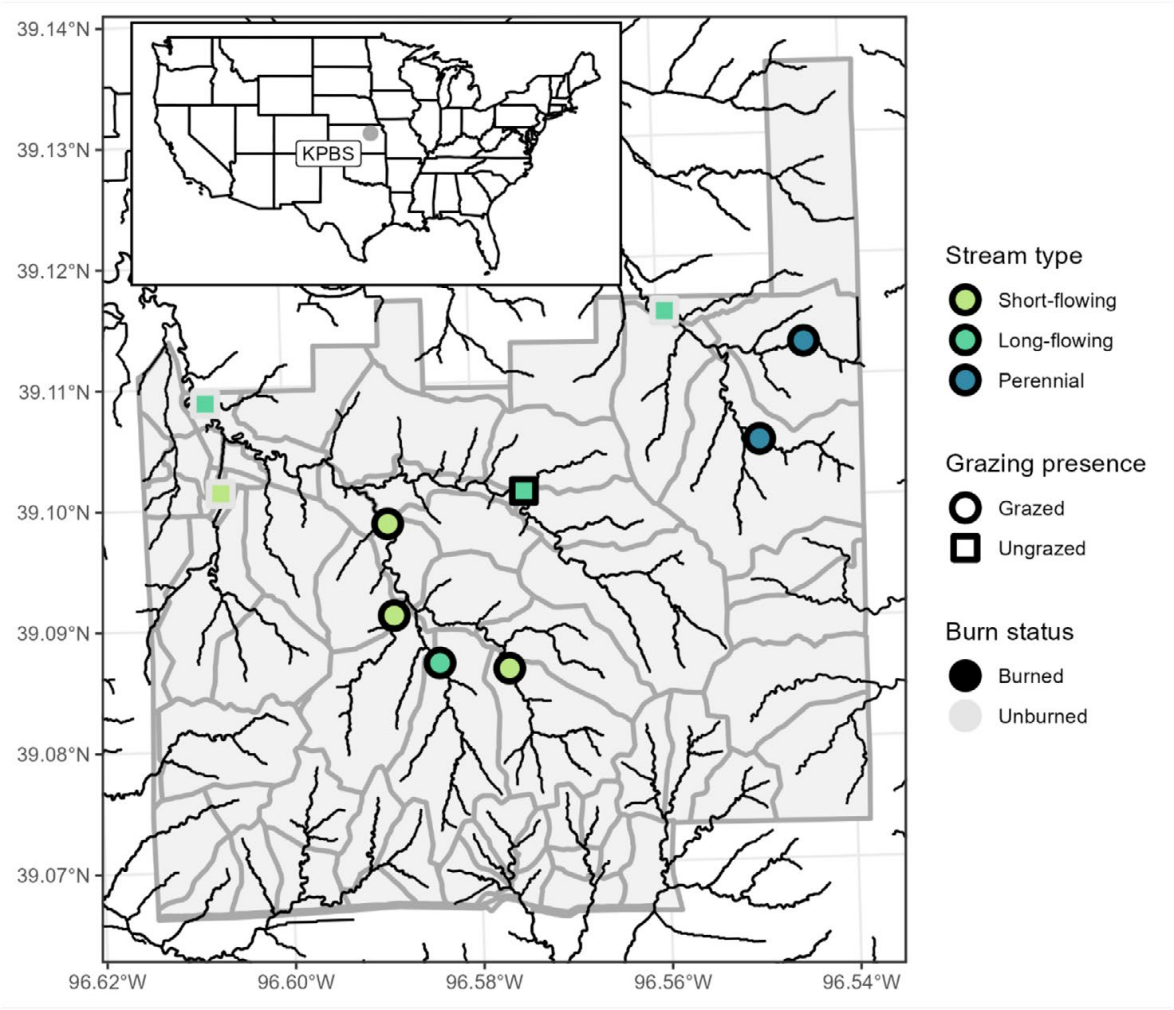
Sampling locations on Kings Creek and Shane Creek stream networks located in Konza Prairie Biological Station (KPBS; gray‐shaded region), Kansas, USA. Gray boundaries represent sub‐watershed management areas.

Kings Creek and Shane Creek watersheds are located within KPBS (Figure 1). Segments of Kings Creek and Shane Creek stream networks are non‐perennial. In these networks, stream drying generally occurs during periods of low precipitation. The complex karstic geology of the region is characterized by layers of high hydraulic conductivity limestone and low hydrologic conductivity mudstone layers (Mast and Turk 1999; Fitzgerald and Bohall 2016; Vero et al. 2018). This geologic composition allows for a small number of areas to hold water year‐round. With substrate of high permeability, there can be an increase in groundwater‐surface water interactions, seeps, or drains (Sophocleous 2002). Seeps and groundwater interactions create microhabitats that support unique groups of species and provide an important water source during dry periods (Mast and Turk 1999).

Sub‐watersheds within Kings Creek and Shane Creek watersheds differ in experimental grazing and prescribed burn treatments. Each sub‐watershed is grazed by native bison, by non‐native cattle, or is ungrazed. Sub‐watersheds are either unburned or burned at varying (1, 2, 3, 4, or 20 years) time intervals.

### 2.2 Data Collection

We established 10 sampling sites in Kings Creek and Shane Creek watersheds. Sites were selected with the intention of having an equal number of perennial and non‐perennial sites, although drying varies from year to year. Four sites were located in sub‐watersheds with bison, two in sub‐watersheds with cattle, and four in sub‐watersheds with no grazing. Additionally, seven of the sites were located in sub‐watersheds with prescribed burns at one (three sites), two (one site), three (two sites), and four (one site) year intervals. Three sites are in unburned sub‐watersheds. At each site, we established a 150 m stream reach as the study area and deployed a Stream Temperature, Intermittency, and Conductivity (STIC) logger every 30 m along the reach in the deepest part of the stream (five STICs per site, 50 STICs total) (Chapin et al. 2014). Each STIC recorded the presence or absence of water every 30 min. At two sites, STICs were repeatedly dislodged due to high flows. At these locations, we installed trail cameras to replace the STICs (four total across the two sites), which photographed the location daily. STIC and trail camera data were aggregated at each site to determine the presence or absence of water at a site at a daily time step. A site was considered “wet” on a given day if more than half of the recordings for that day recorded the presence of water. We then calculated surface water permanence at each site as the porportion of wet days for the time period spanning 1 year preceding the first sampling event through the second sampling event (June 4, 2020–May 23, 2022). We categorized sites by stream type based on surface water permanence values (Bunting et al. 2021). Sites where surface water permanence was equal to 1.0 were considered “perennial.” We considered sites where surface water permanence exceeded 0.5 but was less than 1.0 to be “long‐flowing,” and sites where surface water permanence was less than 0.5 to be “short‐flowing.”

We sampled aquatic macroinvertebrates once each in 2021 and 2022 during the wettest part of the year (May–June). At each site, we collected both benthic and edge macroinvertebrate samples. We collected benthic and edge samples because we thought they may be differently impacted by disturbances related to grassland management due to the proximity of edge communities to the terrestrial landscape. To collect the benthic macroinvertebrate samples, we collected and composited 11 benthic subsamples from across each 150 m reach, working in the downstream to upstream direction. We altered the position of the subsample between the left, center, and right portions of the channel (25%, 50%, and 75% of the wetted channel width, respectively). Each subsample was collected using equipment most appropriate for the habitat, based on stream width and depth (surber sampler, mini‐surber sampler, or D‐net). Each subsample collection area was one net‐width long and wide. If a subsample location was dry, no subsample was taken. At each reach, we recorded the total number of subsamples collected and the device used for sampling, allowing for the calculation of the total area sampled. We then combined the benthic subsamples from a site into a single composite sample and processed it in the field to remove coarse debris and sediments. We collected edge samples by sweeping a D‐net along the reach's wetted edge in riparian‐adjacent microhabitats, targeting submerged vegetation, roots, rocky stream margins, and overhanging banks. At each site, five sweeps were spaced roughly equally throughout the 150 m reach. Edge samples were not quantitative but had the goal to detect taxa that were missed by the benthic sampling. The benthic composite samples and edge samples were preserved in Nalgene containers filled with 95% ETOH.

In the laboratory, we examined a minimum of 500 macroinvertebrate specimens per sample type per site. After visually assessing specimen densities, we used a Folsom plankton splitter to produce subsamples, or “splits,” that we thought would contain at least 500 specimens. We processed additional splits if there were fewer than 500 specimens. For samples with less than 500 specimens total, we processed the entire sample collected. We identified specimens using available keys (Stewart and Stark 1988; Wiggins 1996; Thorp and Covich 2009; Merritt et al. 2019; Tennessen 2019). Aquatic invertebrates were identified to the genus‐level whenever possible, but otherwise to the family‐ or order‐level for challenging taxa (e.g., Oligochaeta) or early instar individuals. Whenever possible, we associated early instar individuals with later instar individuals identified to genus‐level, but in samples without any later instar individuals, identifications of early instar individuals without diagnostic characteristics were left at the family level. Before analysis, we adjusted counts for each taxon based on the split processed in the lab to estimate the total number of individuals in an entire sample. For benthic samples, we also adjusted counts for each taxon by dividing them by the total area sampled during benthic sampling at each reach.

### 2.3 Data Analysis

We quantified aquatic macroinvertebrate community diversity using two metrics: taxonomic richness and the Shannon diversity index. Taxonomic richness refers to the number of taxa present in a sampling area. The Shannon diversity index is a measure of taxa richness and evenness within a community, where a larger number indicates greater diversity. We used the R package “vegan” to calculate diversity metrics (Oksanen et al. 2001). We calculated richness and Shannon diversity for benthic samples. For edge samples, we only calculated richness because the samples were not quantitative. Therefore, we had three metrics of community diversity: benthic richness, benthic Shannon diversity, and edge richness.

We used linear mixed‐effects models to determine the influence of variables related to stream type, grazing presence, and prescribed burn status on aquatic macroinvertebrate community diversity. We considered the following variables as fixed effects: stream type (perennial, long‐flowing, short‐flowing), grazing presence (grazed, ungrazed), burn status (burned, not burned), and sampling year (2021, 2022). We chose to include sampling year as a fixed effect because it captures interannual variations in hydrology and other environmental conditions (e.g., water temperature). We included watershed as a random effect to account for spatial nestedness of sampling sites. Models were fit using the R package “lme4” (Bates et al. 2025). We evaluated the significance of fixed effects in the models with Wald's *χ* ^2^ tests using the R package “car” (Fox et al. 2024). While we were interested in exploring additional variables associated with the land management treatments, such as grazer type and burn frequency, our sample size did not allow for the analysis of these additional factors.

We also explored variability in aquatic macroinvertebrate community composition due to stream type, grazing presence, burn status, and sampling year. For the benthic samples, we created distance matrices using Bray–Curtis dissimilarity matrices of raw abundance data. For the edge samples, we used Jaccard dissimilarity matrices of presence‐absence data, due to the non‐quantitative nature of the samples. Using the distance matrices, we tested for differences among samples using PERMANOVA. We also used the distance matrices to run Principal Coordinate Analyses (PCoAs) in order to visualize differences in community composition in relation to variables that were found to explain variability in community composition. We used the R package “vegan” (Oksanen et al. 2001) to create distance matrices and conduct PERMANOVAS and “ape” (Paradis et al. 2002) to run PCoAs. All analyses were done R (v4.4.1; R Core Team 2024).

## 3 Results

In total, 62,400 individuals from 135 taxa were collected and identified in benthic samples. Edge samples were composed of 20,533 individuals from 138 taxa. We collected 32 taxa unique to the benthic samples, 35 taxa unique to the edge samples, and 103 taxa that were found in both habitat samples.

Surface water permanence varied among sites. Surface water permanence at sites during the study period ranged from 0.29 to 1.0. On the basis of surface water permanence values, four sites were found to be short‐flowing, four sites were long‐flowing, and two sites were perennial. Streamflow and drying extent during sampling varied between sampling years. Streamflow was noticeably lower during sampling in 2022 than in 2021 at many sampling sites. For example, a site that was flowing during sampling in 2021 was almost completely dry during sampling in 2022 with only sporadic, isolated pools remaining. As a result, no edge sample was collected from that site in the second year.

**FIGURE 2:**
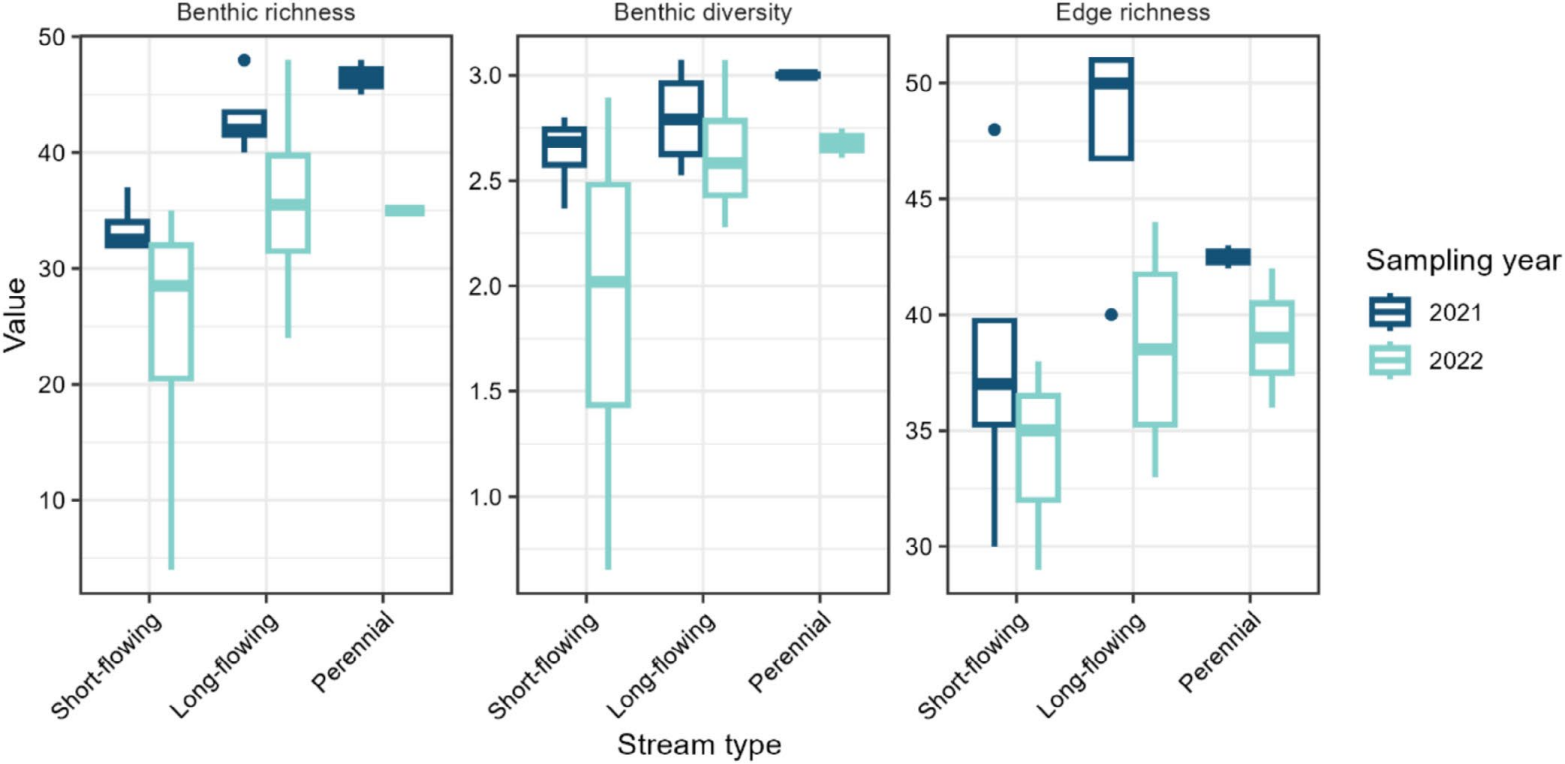
Effects of stream type and sampling year on benthic richness, benthic diversity, and edge richness. Benthic richness and edge richness, but not benthic diversity, varied significantly with stream type and sampling year (Tables 1 and 2).

Macroinvertebrate community diversity varied with stream type and sampling year. Linear mixed effects models showed that benthic taxonomic richness varied with stream type (*χ* ^2^ = 8.08, *p* < 0.05) and with sampling year (*χ* ^2^ = 6.31, *p* < 0.05), but not with grazing presence nor burn status (Table 1). Benthic taxonomic richness was lowest in short‐flowing streams and greatest in perennial streams. Additionally, benthic richness was lower in 2022 than in 2021 (Figure 2). Linear mixed effects models showed that edge taxonomic richness varied with stream type (*χ* ^2^ = 7.00, *p* < 0.05) and sampling year (*χ* ^2^ = 6.33, *p* < 0.05), but not with grazing presence nor burn status (Table 2). The taxonomic richness of edge macroinvertebrate communities was greatest in long‐flowing streams and lowest in short‐flowing streams. Richness was also lower in 2022 than in 2021 (Figure 2). Benthic macroinvertebrate Shannon diversity did not vary with stream type, sampling year, grazing presence, nor burn status (Table 1).

**TABLE 1:** Summary of linear mixed effect model results explaining variation in benthic macroinvertebrate taxonomic richness and diversity with stream type, grazing presence, burn status, and sampling year. Watershed was included as a random effect in the models.

	Benthic richness				Benthic diversity			
	Estimate	Std. error	*χ* ^2^	*p*	Estimate	Std. error	*χ* ^2^	*p*
Intercept	42.147	4.054			2.1907	0.2981		
Fixed effects								
Stream type			8.081	0.018			5.210	0.074
Short‐flowing	—	—			—	—		
Long‐flowing	10.441	4.761			0.5762	0.2898		
Perennial	11.485	5.050			0.4788	0.3074		
Grazing presence			0.148	0.701			0.088	0.787
Ungrazed	—	—			—	—		
Grazed	−2.794	7.272			0.1312	0.4427		
Burn status			0.520	0.471			0.377	0.539
Unburned	—	—			—	—		
Burned	4.853	6.733			0.2517	0.4098		
Sampling year			6.307	0.012			3.801	0.05122
2021	—	—			—	—		
2022	−9.000	3.584			−0.4253	0.2181		

**TABLE 2:** Summary of linear mixed effect model results explaining variation in edge macroinvertebrate taxonomic richness with stream type, grazing presence, burn status, and sampling year. Watershed was included as a random effect in the model.

	Estimate	Std. error	*χ* ^2^	*p*
Intercept	42.147	4.054		
Fixed effects				
Stream type			6.996	0.030
Short‐flowing	—	—		
Long‐flowing	9.355	3.643		
Perennial	4.463	3.479		
Grazing presence			0.505	0.477
Ungrazed	—	—		
Grazed	3.641	5.124		
Burn status			0.009	0.926
Unburned	—	—		
Burned	−0.430	4.647		
Sampling year			6.334	0.012
2021	—	—		
2022	−6.413	2.548		

**FIGURE 3:**
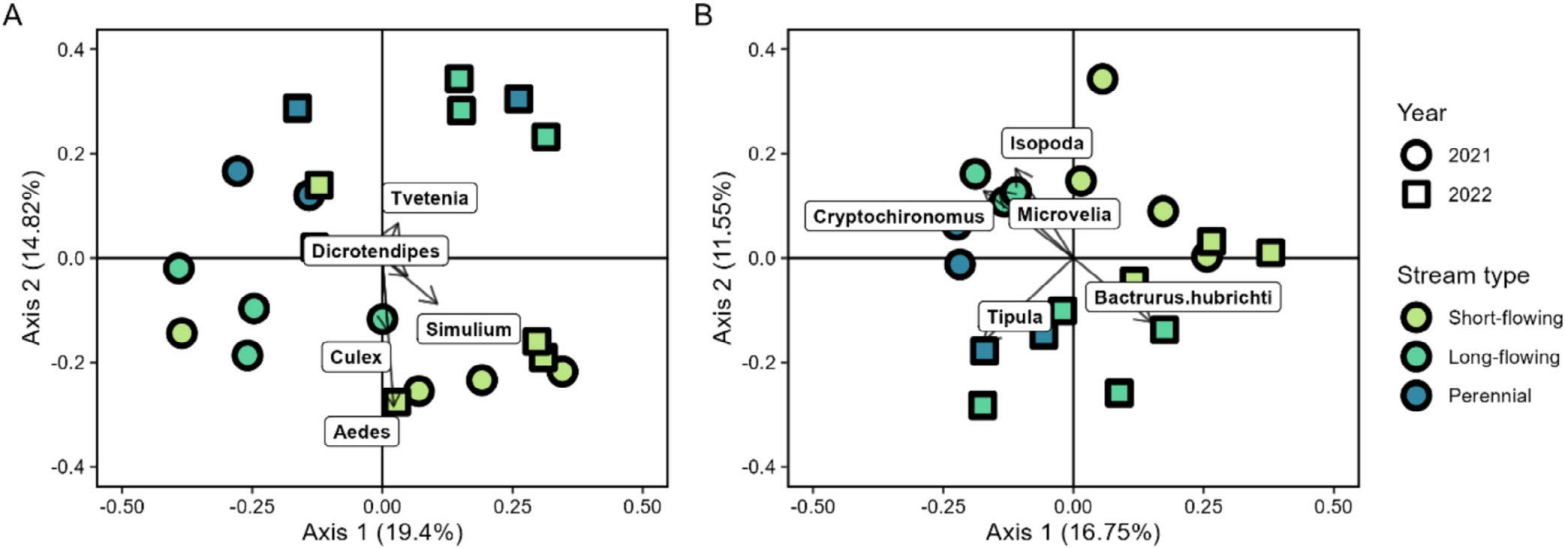
Principal coordinate analyses of (A) benthic and (B) edge macroinvertebrate communities. Arrows represent the influence of the taxa driving differences in community compositions. Only the five most important taxa are shown to improve readability. PERMANOVAs show that both benthic and edge community composition are driven by sampling year and stream type.

Variation in community composition of samples was also driven by stream type and sampling year. PERMANOVA analysis showed that the composition of benthic samples varied by stream type (*p* < 0.05, pseudo‐*R* ^2^ = 0.15) and sampling year (*p* < 0.01, pseudo‐*R* ^2^ = 0.12). Edge samples also varied by stream type (*p* < 0.01, pseudo‐*R* ^2^ = 0.20) and sampling year (*p* < 0.01, pseudo‐*R* ^2^ = 0.10). Visual inspection of the PCoA of benthic macroinvertebrate community data shows separation among samples collected from different stream types and between samples collected in different years (Figure 3A). The five taxa with the greatest loadings were *Aedes, Simulium, Culex, Tvetenia*, and *Dicrotendipes*. Similar to the benthic samples, visual inspection of the PCoA of edge sample data also showed clear separation between sampling years and among stream types. However, different taxa were found to drive differences among edge samples. In the PCoA of edge samples, the five taxa with the greatest loadings were *Tipula*, *Cryptochironomus*, *Microvelia, Bactrutus hubrichti*, and Isopoda (Figure 3B). Notably, *Bactrutus hubrichti* was only collected in 2022, while the other four taxa were collected during both sampling years.

## 4 Discussion

To examine the impacts of the stream drying and land management treatments, we collected benthic and edge macroinvertebrate samples from sites that varied by surface water permanence, grazing presence, and burn status. We found that macroinvertebrate community diversity and composition were influenced by sampling year and surface water permanence (supporting H1), and did not find evidence of the influence of grazing or prescribed burns (not supporting H2). Benthic and edge taxonomic richness tended to increase with surface water permanence and were greater in the first year of sampling, when streamflow and surface water extent were greater preceding and during sampling. The consistent finding of variables related to streamflow driving macroinvertebrate community diversity and composition highlights the important role of streamflow regime in structuring stream ecosystems, and that it potentially overshadows impacts of potential disturbances, such as grazing and fire.

Our first hypothesis, that diversity would increase with surface water permanence, was largely supported. Our findings are consistent with previous research (Datry 2012; Datry, Larned, Fritz, et al. 2014; Schriever et al. 2015). Studies have repeatedly found that perennial sites have more diverse macroinvertebrate assemblages than nonperennial sites. In non‐perennial streams, greater surface water permanence has been consistently correlated with greater macroinvertebrate richness and diversity (Grimm et al. 2013; Giam et al. 2017). Many taxa are not able to withstand conditions associated with drying, including frequent loss of critical habitats and dynamic environmental conditions, and therefore cannot persist in non‐perennial streams (Bogan et al. 2017). While the relationship between surface water permanence and macroinvertebrate diversity is well established, several studies have found similar macroinvertebrate richness in stream with varying surface water permanence (Fritz and Dodds 2004; Leigh et al. 2016; Kelso and Entrekin 2018). In these cases, factors other than drying, such as floods, are attributed with having a strong control on macroinvertebrate richness.

**FIGURE A1:**
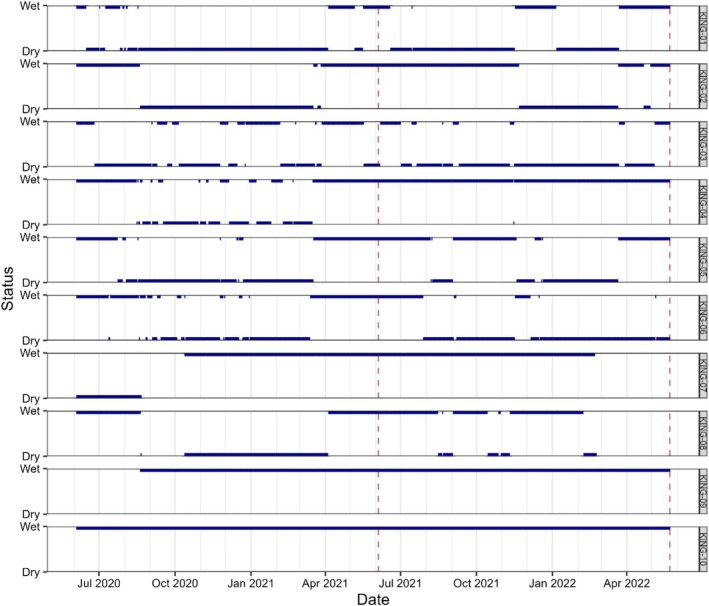
Wet/dry status of the 10 sampling sites over the study period. Red dashed lines indicate sampling dates.

We also found that macroinvertebrate richness varied with sampling year. While there were likely numerous environmental factors that contributed to interannual variation in the macroinvertebrate samples, our observations in the field suggest differences in precipitation and streamflow, specifically in the weeks preceding sampling, were an important factor. In the weeks preceding sampling, there was more rainfall in 2021 than in 2022, resulting in increased streamflow preceding and during sampling in 2021 (U.S. Geological Survey 2016; Figure A1). Benthic and edge taxonomic richness were both greater in samples from 2021 than those from 2022. Reduced diversity in 2022 can be attributed to reduced streamflow that year. Short‐term impacts of low flow events on stream macroinvertebrates are commonly observed (Miller and Golladay 1996; Fritz and Dodds 2004; Acuña et al. 2005; Bogan et al. 2015). As flow recedes, taxa may be lost as they are no longer able to meet their needs due to factors including loss of critical habitats, reduced oxygen levels, and increased temperatures (Boulton 2003). Recolonization tends to occur quickly following low flow and drying events, so impacts on aquatic communities tend to be short‐term (Miller and Golladay 1996; Bogan et al. 2015). In our analysis, stream type is a measure of long‐term drying patterns and sampling year is a measure of short‐term drying patterns. Both play an important role in structuring stream communities.

In addition to differences in diversity, we identified differences in community composition among stream types and sampling years. Taxonomic differences in community composition among streams with differing drying patterns are inconsistently observed. When differences are documented, they can often be explained by taxa with traits associated with resistance and resilience to drying events, such has aerial respiration, multivoltinism, armoring, desiccation resistance, and high dispersal ability (Bonada et al. 2007; Clarke et al. 2010; Bogan et al. 2013; Kelso and Entrekin 2018; Stubbington et al. 2019; Siders et al. 2025). The differences in community composition among stream types in our study reflected these patterns. Additionally, the occurrence of *Bactruruus hubrichti* was notable because it was found only in edge samples collected in 2022. That *B. hubrichti* , which exists in karstic groundwater habitats, was found exclusively in samples collected in 2022 reflects the reduced precipitation and streamflow during and preceding sampling, and aligns with past research that showed that the number of amphipods per liter collected from a Konza Prairie spring was greatest during periods of low discharge (Edler and Dodds 1996). Whether this reflects affinities of the amphipod or is the result of a dilution effect is unclear.

Our study did not provide evidence of any impact of grazing presence or prescribed burns on aquatic macroinvertebrate communities. Our second hypothesis was therefore not supported. A previous exploratory study of grazing impacts on stream water quality in Konza Prairie Biological Station (KPBS) did not find differences in suspended sediment or nutrient concentrations between grazed and ungrazed sub‐watershed, lending support to our finding of similar macroinvertebrate community diversity and composition in grazed and ungrazed sub‐watersheds (Larson et al. 2013). However, previous studies at KPBS have documented impacts of fire presence on water quality. Dodds et al. (1996) found that nitrate and total N concentrations in surface water decreased as the length of time since last fire increased, while Larson et al. (2013) reported that fire temporarily reduced both total N and total P. The contrasting results of these two studies that both took place within KPBS highlight the high variability of stream responses to fire, and the need for further studies to explain the variability.

Outside of KPBS, studies documenting impacts of grazing and burns on stream macroinvertebrates report mixed findings (Krall and Roni 2023; Erdozain et al. 2024). A variety of factors likely modulate the impacts of grazing and prescribed burns on stream ecosystems. For example, the timing of burns and grazing are thought to be influential, altering soil erosion and runoff (Jourdonnais and Bedunah 1990; Brye et al. 2002; Silver and Vamosi 2012; Williams et al. 2012). Burn intensity, extent, stream size, and precipitation are also thought to mediate the impact of fire on macroinvertebrate communities (Minshall 2003). These additional factors potentially impacted the results of our study. Further, it is also possible that there are interactions between hydrology and land management treatments occurring. For example, bison may prefer watersheds with higher streamflow and greater flow permanence (Larson et al. 2013). With our limited sample size, we were not able to capture these complexities and their potential ecological consequences in our analyses. We acknowledge that our study is therefore exploratory in nature. However, this study builds on the previous research that has taken place in KPBS that examined impacts of grazing and burning on stream water quality by being the first that we are aware of to examine potential impacts on stream macroinvertebrate communities within the biological station. Disentangling the impacts of the co‐occurring stressors of stream drying and grassland management is especially important in the Great Plains where non‐perennial streams are abundant. Future studies will be needed to more fully explain the impacts of grazing and prescribed burns on stream communities in KPBS.

Our study highlights the role of stream drying in shaping stream communities, even in the context of varying grassland management. While there is opportunity for future work to further explore impacts of stream drying, grazing, and prescribed burns on stream communities, our findings suggest that in prairie streams there are benefits to protecting the natural streamflow regime even when potential co‐occurring disturbances are present. Climate change and increased human demand for water both contribute to increased stream drying (Marshall et al. 2010; Price et al. 2021). In the central Great Plains, and many other regions, the temporal and spatial extents of stream drying are increasing (Zipper et al. 2021), a trend that is expected to continue into the future (Chatterjee et al. 2018). When changes to stream drying patterns occur, we should expect changes to stream ecosystems (Perkin et al. 2017; Hopper et al. 2020; Malish et al. 2023; Datry et al. 2023). The implementation of water management strategies that incentivize reduction in water use or establish environmental flows can protect stream ecosystems (Gillespie et al. 2015; Zamani Sabzi et al. 2019; Pander et al. 2019; Wineland et al. 2022). Our findings suggest that protecting streamflow will benefit stream communities across variable landscapes.

## Author Contributions

**Olivia Tow:** formal analysis (equal), visualization (equal), writing – original draft (equal), writing – review and editing (equal). **Megan C. Malish:** conceptualization (equal), formal analysis (equal), investigation (equal), supervision (equal), visualization (equal), writing – original draft (equal), writing – review and editing (equal). **Stephen C. Cook:** investigation (equal), writing – review and editing (equal). **Michael T. Bogan:** data curation (equal), funding acquisition (equal), investigation (equal), project administration (equal), writing – review and editing (equal). **Daniel C. Allen:** funding acquisition (equal), investigation (equal), project administration (equal), supervision (equal), writing – review and editing (equal). **Thomas M. Neeson:** funding acquisition (equal), project administration (equal), supervision (equal), writing – review and editing (equal).

## Funding

This work was supported by Division of Environmental Biology (DEB‐1802766, DEB‐2207680).

## Conflicts of Interest

The authors declare no conflicts of interest.

## Data Availability

Data and code associated with this manuscript are available at https://doi.org/10.17605/OSF.IO/NK6J3
